# Transferable Machine Learning Interatomic Potential for Bond Dissociation Energy Prediction of Drug-like Molecules

**DOI:** 10.1021/acs.jctc.3c00710

**Published:** 2023-12-18

**Authors:** Elena Gelžinytė, Mario Öeren, Matthew D. Segall, Gábor Csányi

**Affiliations:** † Engineering Laboratory, University of Cambridge, Trumpington Street, Cambridge CB2 1PZ, U.K.; ‡ Optibrium Limited, Cambridge Innovation Park, Denny End Road, Cambridge CB25 9GL, U.K.

## Abstract

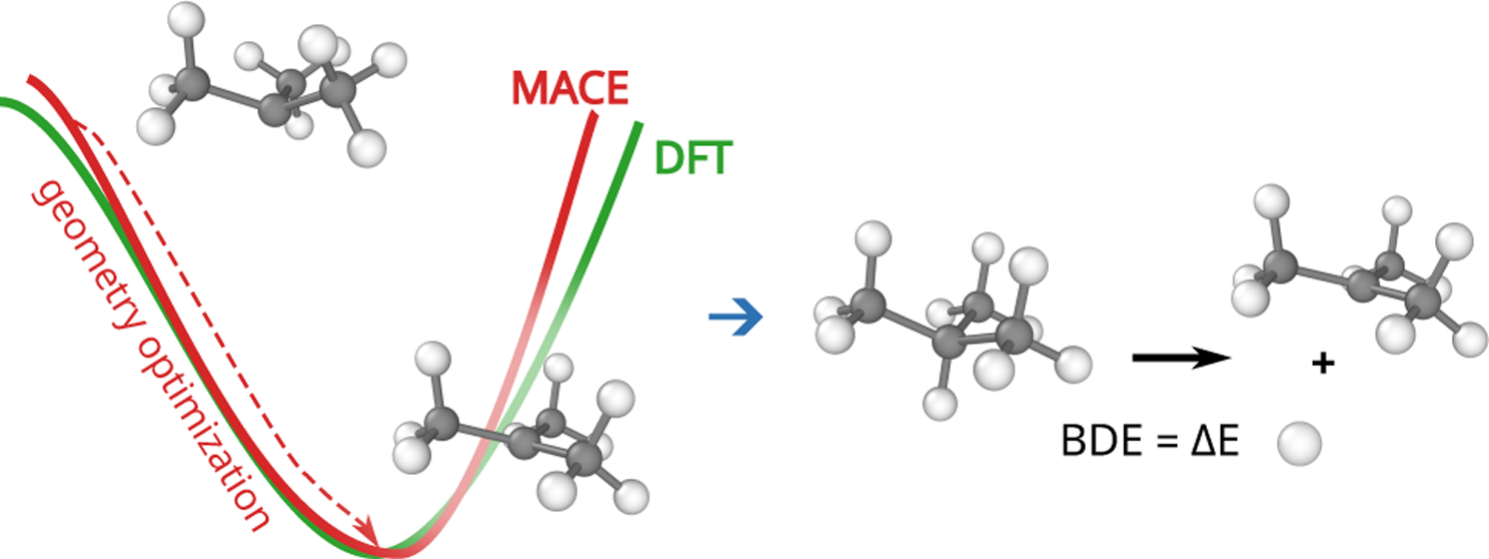

We present a transferable MACE interatomic potential that is applicable to open- and closed-shell drug-like molecules containing hydrogen, carbon, and oxygen atoms. Including an accurate description of radical species extends the scope of possible applications to bond dissociation energy (BDE) prediction, for example, in the context of cytochrome P450 (CYP) metabolism. The transferability of the MACE potential was validated on the COMP6 data set, containing only closed-shell molecules, where it reaches better accuracy than the readily available general ANI-2x potential. MACE achieves similar accuracy on two CYP metabolism-specific data sets, which include open- and closed-shell structures. This model enables us to calculate the aliphatic C–H BDE, which allows us to compare reaction energies of hydrogen abstraction, which is the rate-limiting step of the aliphatic hydroxylation reaction catalyzed by CYPs. On the “CYP 3A4” data set, MACE achieves a BDE RMSE of 1.37 kcal/mol and better prediction of BDE ranks than alternatives: the semiempirical AM1 and GFN2-xTB methods and the ALFABET model that directly predicts bond dissociation enthalpies. Finally, we highlight the smoothness of the MACE potential over paths of sp^3^C–H bond elongation and show that a minimal extension is enough for the MACE model to start finding reasonable minimum energy paths of methoxy radical-mediated hydrogen abstraction. Altogether, this work lays the ground for further extensions of scope in terms of chemical elements, (CYP-mediated) reaction classes and modeling the full reaction paths, not only BDEs.

## 1 Introduction

Due to their computational efficiency, empirical force fields play an indispensable role in computational chemistry, particularly in biomolecular simulations and computer-aided drug design.^1,2^ The traditional empirical force field approach assigns atom types based on an atom’s bonding pattern and chemical element and represents the potential energy surface with atom-type-specific bonded harmonic terms for bonds, angles, and torsions as well as nonbonded Lennard-Jones and Coulombic terms.^3−5^ Typically, the parameters are fitted to reproduce experimentally observed structural, dynamical, and electrostatic properties or data from higher-level electronic structure calculations, for example, partial charges, torsional profiles, and vibrational spectra of small molecules.^5−7^ One important application of empirical force fields is free-energy calculations of protein–ligand interactions.^6,8−10^ Protein structure descriptions are generally reliable and transferable^11^ because of small bond length and angle deviations from typical values over the course of a simulation and due to the well-defined chemical space of canonical amino acids, which exhibit conserved structures among the majority of studied proteins.^12^ However, the chemical space of drug-like molecules is considerably more complex, and the range of reasonably low-energy conformations is wider.^13,14^ As a consequence, the existing small molecule force fields are typically trained on only a fraction of the compounds needed for transferability.^4^ As a result, the aforementioned parameters, such as bond-spring constants, need to be fine-tuned for the force field to accurately represent the potential energy surface and interactions of the particular small molecule of interest in applications that require higher accuracy, such as binding free-energy calculations.^15^ Another application of empirical force fields is conformational analysis, for assessment of the lowest energy and most relevant conformers of small molecules.^16^ In this application, empirical force fields are mainly used as initial structure generation tools, with subsequent refinement needed by semiempirical or ab initio methods.^17−19^ Empirical force field-based conformational searching also plays a significant role in modeling organic reaction mechanisms,^20,21^ even though they are not designed to describe nonequilibrium structures and substantial modifications are necessary if reactivity is to be modeled.^22,23^ To further refine the structures and investigate reactions, ab initio methods are necessary as with conformational searches. Although ab initio methods offer a significant improvement in accuracy over empirical force fields and allow the study of reactive systems, they are mostly too computationally expensive for routine use in, for example, high-throughput virtual screening (e.g., 100s to 1000s of molecules processed in a span of hours) or routine molecular dynamics (MD) simulations of systems with 1000s of atoms.

In recent years, machine learning interatomic potentials (MLIPs) have emerged as successful tools for accurately approximating ab initio potential energy surfaces at a significantly reduced computational cost: roughly milliseconds instead of minutes per single-point evaluation of a typical structure found in this work.^24−28^ MLIPs have found applications in various computational chemistry problems, ranging from highly accurate spectra prediction^29^ long MD simulations of electrolytes,^30,31^ path-integral MD of supramolecular complexes,^32^ to crystal structure prediction,^33^ heterogeneous catalysis,^34,35^ and radical reaction networks.^36^ MLIPs offer an efficiency advantage by avoiding the need to solve the all-electron Schrödinger equation; instead, they predict energy and forces from the atom positions directly. MLIPs are constructed via local environment representations, enabling linear scaling and transferability to structures orders of magnitude larger than those in the training set.^37^ Unlike empirical force fields, MLIPs do not rely on a predefined functional form and exhibit flexibility in representing complex potential energy surfaces.

One of the primary tasks of fitting MLIPs is to collect appropriate structures for the reference database, which determines the domain of applicability of the model. For example, some successful MLIPs in materials modeling are fitted to relatively small unit cells containing tens of atoms, enabling MD simulations of up to a hundred thousand atoms for a few nanoseconds.^37,38^ The situation is equivalent for MLIPs for small organic molecules, where the chemical space of potential compounds is too vast for the training set to encompass every possible compound.^14,39^ Therefore, MLIPs based on local environments are expected to extrapolate to novel chemical entities if the training database contains a sufficient number of atomic environments that are similar to those in the new compound. Efforts to achieve transferability in small molecule MLIPs are evident in existing benchmark data sets^40−43^ and general purpose transferable MLIPs, such as the ANI series.^28,44,44^ However, applications of these MLIPs beyond near-equilibrium closed-shell structures remain limited.

**Figure 1:**
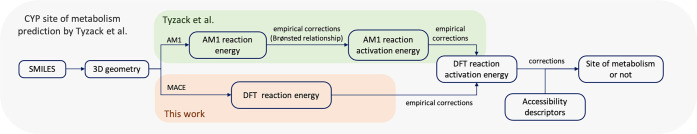
Framework to predict Cytochrome P450 sites of metabolism described by Tyzack et al.^45^ for the context of where transferable MLIPs for reaction (activation) energy prediction could offer an improvement.

High-throughput prediction of cytochrome P450 (CYP) metabolism is an example where both transferability and applicability to open-shell structures are required.^45^ CYPs are a family of heme-containing enzymes that account for approximately 90% of the phase I drug metabolism.^46^ Consequently, computational tools capable of predicting sites of metabolism are instrumental in drug development campaigns. Unlike most enzymes, where the active site is adapted for a specific substrate, the important CYP isoforms exhibit low specificity toward their substrates, with substrate reactivity playing a significant role in determining the sites of metabolism. This motivates a ligand-based approach, where the influence of the active site environment is not taken into account mechanistically. Such an approach, as described by Tyzack and colleagues^45^ is illustrated in Figure 1. In their work, the prediction of metabolic lability is based on the estimation of ligand reaction activation energy along with CYP isoform-specific corrections based on site accessibility. The reaction activation energy is predicted from semiempirical AM1 reaction energy and two subsequent corrections. The first correction relies on a Brønsted relationship to estimate the AM1 reaction activation energy from the AM1 reaction energy, while the second correction addresses systematic errors associated with the AM1 method with DFT as a reference. DFT-accurate transferable MLIPs have the potential to replace AM1 in this approach, improving the accuracy and speed of high-throughput reaction-energy or even reaction-activation energy prediction, as highlighted in the lower branch of Figure 1.

Reaction pathways catalyzed by CYPs proceed via radical intermediates, necessitating methods capable of treating open-shell structures for studying small molecule metabolism. However, there are few methods to study radicals without invoking the full electronic structure treatment, which is too computationally expensive for high-throughput predictions. Most notable are semiempirical methods like AM1^47^ or GFN2-xTB,^48^ which are the simplest form of electronic structure theory, with integral approximations and parametrization fitted to experimental data or higher-level ab initio results.^49,50^ As such, they are in between ab initio methods and empirical force fields, offering faster computation but lower accuracy compared to the former and greater transferability across a wider range of chemistry problems at a higher computational cost than the latter. A few empirical force fields have been created specifically for modeling reactions and open-shell structures, particularly the ReaxFF potentials.^51^ While various ReaxFF parametrizations have been used to study a wide range of challenging problems,^22^ there is still room for improvement to better match ab initio potential energy surfaces.^52^

To address this specific need, we present a proof-of-concept work introducing a transferable and computationally efficient MACE interatomic potential that encompasses drug-like molecules and radical species. MACE, an equivariant message-passing neural network (MPNN), demonstrates exceptional speed, accuracy and data efficiency, surpassing existing methods on a range of benchmark tasks.^53,54^ Our training data set is derived from the ZINC database^55^ and consists of drug-like molecules containing carbon, hydrogen and oxygen ([C,H,O]) atoms, as well as derived sp^3^ carbon radicals. The choice to limit this study to compounds containing [C,H,O] chemical elements was motivated by smaller chemical space and easier development of the models, with the intention to subsequently expand the scope of chemical elements and CYP-catalyzed reaction mechanisms to possibly integrate these models in the site of metabolism prediction framework illustrated in Figure 1. We have tested the model’s transferability on CYP substrates and the MLIP benchmark COMP6 data set.^41^ The results demonstrate that our model achieves better accuracy than a general ANI-2x potential on a relevant part of the COMP6 data set while extending the area of applicability of the model to sp^3^ carbon radicals.

In this study, we apply our MACE potential to predict bond dissociation energy (BDE), similarly to how the AM1 method is used in the CYP metabolism prediction described by Tyzack et al.^45^ We focus on the hydrogen abstraction reactions facilitated by CYPs, whose rate-limiting step is characterized by the aliphatic C–H BDE. It is possible to choose BDE to be the target and to be predicted directly by the ML model, for example, similarly to how st. John et al.^56^ were modeling bond dissociation enthalpies. In such a case, a database of BDEs is computed with an ab initio method, and an ML model is created to predict those BDEs based on, for example, the structures’ 2D representations (SMILES strings). Our approach instead chooses to retain the mechanistic aspect of computing the BDEs and instead uses an ML force field as a computationally efficient DFT alternative to drive the geometry optimization and evaluate the total energies from which the BDE is computed. Therefore, no BDE data has been included in the MLIP training set and, because BDE is an equilibrium property, the training set only includes structures sampled from 500 K MD trajectories and no structures from the full hydrogen abstraction paths. By fitting an ML interatomic potential, rather than predicting the property of interest directly, the model can be additionally applied to compute other (thermodynamic) quantities, such as bond dissociation enthalpies.

Our results show that MACE accurately predicts BDEs, achieving a root-mean-square error (RMSE) of 1.37 kcal/mol on a set of compounds with observed CYP 3A4 isoform sites of metabolism. In addition, MACE’s BDE ranks show a good correlation with the DFT BDE ranks, improving on the predictions of the semiempirical AM1 and GFN2-xTB methods or the graph neural network ALFABET model.^56^ We further test the model’s extrapolation ability by applying it to nonequilibrium structures obtained by dissociating a C–H bond. Finally, we slightly extend the training set of the near-equilibrium isolated closed- and open-shell structures by additionally including examples of reactants and products of multiple hydrogen abstractions by methoxy radical reactions. This modification was enough to find reasonable minimum energy paths (MEPs) with the nudged elastic band method, thus demonstrating the feasibility of extension of the model to calculate the hydrogen abstraction reaction barriers, not only reaction energies.

## 2 Methods

### 2.1 MACE Framework

We chose MACE, an equivariant MPNN framework, which has been shown to perform similarly or better than 11 contemporary MLIPs on the small molecule rMD17 benchmark.^53,57^ Furthermore, MACE has demonstrated high accuracy and extrapolation capacity when compared to NequIP MPNN^58^ on the 3BPA^59^ and AcAc benchmarks.^53,60^ A subsequent study demonstrated MACE’s excellent performance on a wider range of tasks: from improving on a global model’s accuracy on the MD22 data set of large molecules,^32^ to transferability to COMP6 benchmark by fitting to 10% of ANI-1x training set,^41,61^ to improving on the state of the art for many properties of the QM9 quantum chemistry benchmark,^62^ among others, as compared with several contemporary MLIPs.^54^ With respect to the computational efficiency, a reasonably sized MACE model has a latency of 24.3 ms per evaluation—around four times faster than other equivariant MPNN models.^53^

In MACE, atomic structures are treated as graphs, where nodes represent atoms in three-dimensional space, and any two nodes within a cutoff distance are connected. A number of message-passing iterations are performed to update the features associated with each node. The final atomic site energy prediction is read out as a function of all of the node’s states created over the iterations.

Messages in MACE can be of arbitrary body order, constructed in a computationally efficient way based on Atomic Cluster Expansion framework.^63^ As a result, MACE models can reach a given accuracy with fewer message-passing iterations than if conventional 2-body messages were used. Spherical tensors of arbitrary order are used as messages further improving the expressivity of the structure description and thus the model’s performance.^60^

Detailed equations defining MACE architecture may be found in ref (54). Key MACE parameters for the model used in this work are reported in Table S1 of the Supporting Information.

### 2.2 Reference Method

Throughout this study, the B3LYP-D3BJ dispersion corrected functional with the def2-SV(P) basis was employed as a computationally efficient yet reliable reference electronic structure method. B3LYP has demonstrated comparable performance to more expensive hybrid meta-GGA functionals in tasks such as geometry optimization and transition state searches for small organic molecules.^64^ Furthermore, B3LYP is widely used to study CYP metabolism^65^ and is often the basis of metabolism models similar to the ones that motivated our work.^66,67^

In order to calculate the homolytic BDE, both open- and closed-shell structures have been included in the MACE training data set. Unrestricted DFT calculations were used with singlet states (*M*_S_ = 0) for closed-shell structures and doublet (*M*_S_ = 1/2) for open-shell structures. To facilitate convergence, electronic smearing at 5000 K was applied.

### 2.3 Data Sets

A variety of structures from different sources were used to evaluate the performance of the MACE model presented in this study. Table 1 provides a summary of these sources, and additional details are provided in the subsequent subsections. Additionally, distributions of heavy atoms in each of the data sets may be found in Figure S1 of the Supporting Information.

**Table 1:** Various Data Sets Used in This Work^a^

	number of compounds	number of topologies	number of configurations	mean / max # heavy atoms	open-shell structures?	description
ZINC-train	12,985	16,824	16,824	18.9 / 27	yes	drug-like compounds from ZINC database, sampled from 500 K GFN2-xTB or MACE MD
ZINC-test	3028	4642	4642	16.6 /36	yes	different drug-like compounds from ZINC database, sampled from 300 K GFN2-xTB MD
CYP 3A4	60	1177	1177	25.0 /42	yes	CYP substrates with experimentally observed SOMs, sampled from 300 K GFN2-xTB MD
Tyzack 2016	105	315	630	18.6 / 42	yes	CYP substrates from Tyzack 2016, sampled from 300 K GFN2-xTB MD
DrugBank	177	177	2819	22.0 / 57	no	real drug molecules from DrugBank part of COMP6 benchmark
GDB7–9	329	329	7896	7.9 / 9	no	7–9 heavy atom containing nonequilibrium structures from GDB7–9 part of COMP6 benchmark
GDB10–13	449	449	7356	11.4 / 13	no	10–13 heavy atom containing nonequilibrium structures from GDB7–9 part of COMP6 benchmark

a The purpose of the “Number of compounds/topologies/configurations” columns is to give a sense of the diversity of structures in each of the data sets. “Number of configurations” refers to the total number of 3D geometry entries in the data set. Multiple 3D geometries may have the same “topology” or “connectivity”, e.g., different conformers of the butane molecule are classed as having the same “topology”. Finally, the data sets that contain both closed- and open-shell structures may have both, the parent closed-shell molecule and, possibly multiple, open-shell radicals derived from the parent molecule by removing an appropriate hydrogen atom. Because their structures significantly overlap, “Number of compounds” counts the geometries of the parent molecule and derived open-shell structures as the same “compound”. The COMP6 benchmark was first described by Smith & co.^41^ In all cases, only the [C,H,O]-containing structures were used.

#### 2.3.1 Train and Test Sets

**Figure 2:**
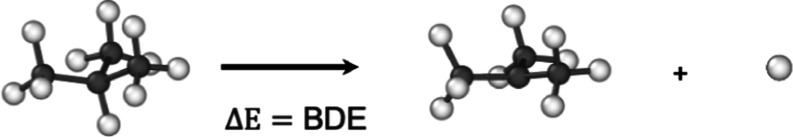
Example of homolytic bond dissociation.

The ZINC database contains over 120 million purchasable organic molecules^55^ which served as a source of diverse compounds for constructing training and testing datasets for MACE. Because BDE calculation necessitates the treatment of radical species (see Figure 2), both closed-shell molecules and derived radicals needed to be included in the training and testing datasets. In this work, we have focused on aliphatic C–H bond dissociation energies and, as a result, only radicals generated by a homolytic cleavage of a sp^3^C–H bond were considered.

To generate the training set, from the “drug-like” subset of the ZINC database we have randomly selected just under 13,000 SMILES strings that contained only carbon, hydrogen, and oxygen chemical elements (see Table 1). From these SMILES strings, 3D conformers of closed-shell molecules were generated by RDKit.^68^ A number of open-shell radical structures, corresponding to the number of sp^3^ hydrogen atoms in the parent closed-shell molecule, were generated for each of the closed-shell molecules by, in turn, removing (deleting) the appropriate hydrogen atom. From this pool of open- and closed-shell structures, nearly 17,000 were selected as the starting geometries for the MD simulations. To generate the final training set, we performed the MD simulation at 500 K and selected a single structure from each trajectory. To obtain the target energy and force values, all of the selected structures were evaluated with the B3LYP-D3BJ/def2-SV(P) reference method described in section Reference Method. An equivalent procedure was used to generate the testing set, started from entirely new [C,H,O]-containing SMILES strings, with the MD simulation run at 300 K temperature. Note that since BDEs are an equilibrium property, in this work we do not include any of the hydrogen abstraction reaction pathway data, only snapshots from the MD trajectories of closed-shell molecules and open-shell radicals. For a more detailed description of the training set generation procedure, see Section I of the Supporting Information.

#### 2.3.2 Cytochrome P450 Test Sets

As part of this study, we evaluated the MACE model’s performance on two sets of compounds relevant to the CYP metabolism. Compounds in the “Tyzack 2016” data set were collected from ref (45), where they have been used to benchmark CYP metabolism models. The compounds in the “CYP 3A4” data set were gathered and curated from publicly available sources that provide detailed information on the experimentally observed substrates (and sites of metabolism) of human cytochrome P450 3A4 isoform. SMILES strings for compounds in the “CYP 3A4” data set are provided in the Supporting Information. BDE predictions were tested on the “CYP 3A4” set of compounds, for which we also provide energy and force component errors. The geometries on which the errors were evaluated were generated using 300 K GFN2-xTB MD simulations, following the same procedure as for the “ZINC-test” data set.

#### 2.3.3 COMP6 Test Set

The “ANI MD”, “DrugBank”, “GDB7–9”, and “GDB10–13” data sets were constructed by selecting structures containing only the [C,H,O] chemical elements from the original COMP6 benchmark.^41^ Unlike the previous data sets, the geometries were taken directly from the original benchmark to enable a more direct comparison with other models tested on this benchmark. To calculate ANI-2x errors, energies and force components were evaluated with ANI-2x and directly compared to the reference values reported in the COMP6 database. To calculate MACE errors, the structures were re-evaluated using the reference method employed to train the MACE model, as described in the Section Reference Method. Therefore, MACE and ANI-2x errors were computed with respect to the DFT reference values, to which the respective method was fitted: B3LYP-D3BJ/def2-SV(P) for MACE and ωB97x/G6-31(d) for ANI-2x.

### 2.4 Data Set Projections

To assess the diversity of the aforementioned data sets qualitatively, in section Data Diversity, we analyze 2D projections of the data and provide additional details in the Supporting Information.

To generate the projections, the smooth overlap of atomic positions (SOAP) descriptor^69^ was evaluated on all of the structures. In addition to the local SOAP descriptor for each atomic site, a global SOAP descriptor is constructed by averaging the atomic neighbor density expansion coefficients over the atomic sites before the rotationally invariant power spectrum is calculated. This procedure yields a description of the global structure that is more informative than a naive average of local SOAP descriptors across the atom sites.^70,71^ All descriptor instances were normalized to have a unit norm.

SOAP descriptors were calculated with the DScribe package^72^ with “average = ‘off’” for “local” and “average = ‘inner’” for “global” SOAP descriptors. SOAP parameters are described in Table S2 of the Supporting Information.

We have used Uniform Manifold Approximation and Projection (UMAP)^73^ to plot these descriptor instances in 2D space. UMAP is a nonlinear projection method that aims to preserve the global structure of the data. More details on UMAP parameters can be found in Supporting Information Section VB.

### 2.5 Software

The RDKit package^68^ (v2023.9) has been used to generate the initial 3D conformers from the SMILES representation. For handling atomic structures, running MD, geometry optimization, and similar operations, we have extensively relied on the Atomic Simulation Environment.^74^ In addition, we used the wfl and ExPyRe Python packages^75^ for running the relevant calculations efficiently across the large number of structures handled in this work. The SOAP descriptors were calculated with the DScribe package.^72^ The reference DFT calculations were performed with the ORCA v5.0.0 software package.^76^ The ANI-2x model was evaluated using the TorchANI package.^77^ We used the Python API (xtb-python.readthedocs.io) to evaluate GFN2-xTB properties.

## 3 Results

### 3.1 MACE Performance and Transferability

As a first step, to probe the MACE model’s transferability, we have tested it on a range of different data sets as described in Section Data Sets and Table 1.

The MACE energy and force component errors on these data sets as well as ANI-2x errors on the COMP6 data set are reported in Table 2. First, MACE errors are comparable across the different data sets, which demonstrates the transferability of MACE over a wide range of structures represented in each of these data sets. The poorer performance on “GDB” data sets may be explained by the fact that the ZINC training set was constructed to specifically contain drug-like compounds, as loosely motivated by the context of drug metabolism, whereas the original “GDB” data sets were assembled to cover all structures with up to 13 heavy atoms.^39,78^ Larger errors on “GDB” data sets may also be explained by the fact that these compounds are most dissimilar to the compounds in the training set, as discussed in the following section.

**Table 2:** RMSEs on Different Data Sets^a^

	MACE		ANI-2x	
	E	F	E	F
ZINC-test	0.05	0.91		
CYP 3A4	0.04	1.20		
Tyzack 2016	0.06	1.24		
DrugBank	**0.05**	**1.37**	0.07	3.09
GDB7–9	0.12	**1.72**	**0.07**	2.20
GDB10–13	0.14	**3.40**	**0.12**	3.97

a Energy RMSE (“E”) is measured in kcal/mol/atom, and force component RMSE (“F”) in kcal/mol/Å. MACE RMSEs are calculated with respect to the same DFT reference on which MACE was trained on. ANI-2x RMSE is calculated by comparing ANI-2x predictions with values provided in the COMP6 database, both of which correspond to ωB97x/6-31G(d) calculated with Gaussian09.^41^ Both MACE and ANI-2x are evaluated on the same set of structures, as described in Table 1.

When compared with ANI-2x on the COMP6 benchmark, MACE has lower energy errors on the “DrugBank” subset and lower force component errors on all of the subsets. This is particularly significant because in addition to closed-shell molecules which may be evaluated with ANI-2x, MACE is applicable to a much larger space of sp^3^ carbon radicals that may be derived from these closed-shell molecules. In addition, MACE achieves these low errors on the COMP6 subset with only 17,000 structures in its training set.

**Figure 3:**
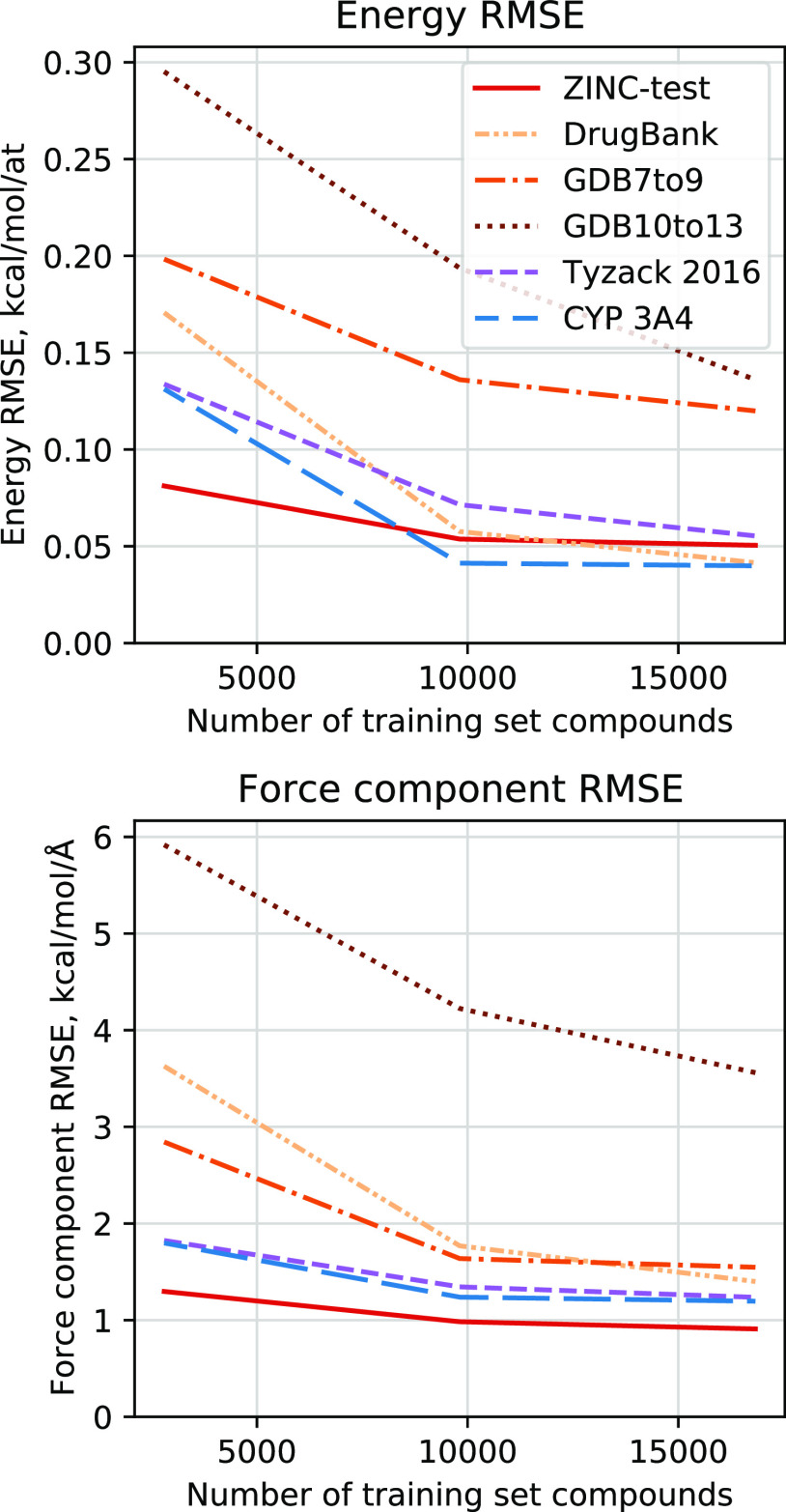
MACE learning curves.

**Figure 4:**
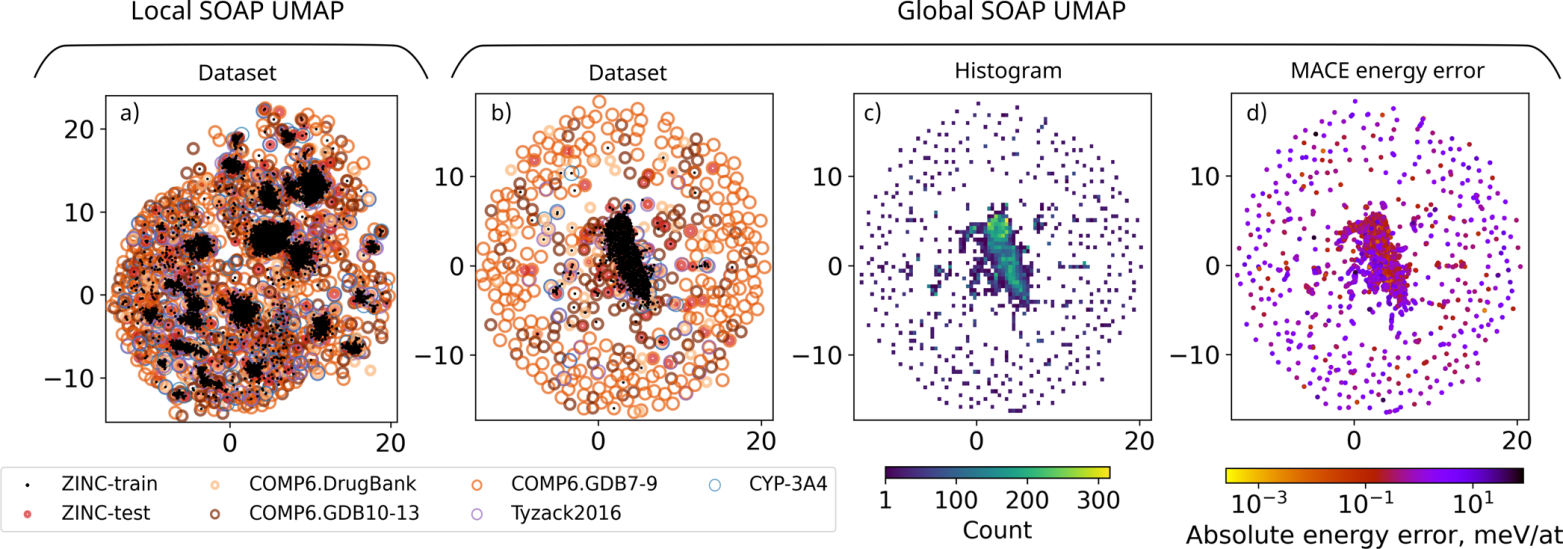
Projections of training and testing sets. (a) UMAP of local SOAP representation, color-coded by data set type; (b) UMAP of global SOAP representation, color-coded by data set type; (c) histogram of global SOAP data in UMAP; (d) UMAP of global SOAP, color-coded by MACE error, with only compounds in the test sets shown. For clarity, in parts (a) and (b), data points were sparsified within each data set to avoid significant overlap. Complementary histograms reflecting data density are provided in the Supporting Information. In (d), data points are plotted in ascending order of error; figures with reversed order are included in Figure S4 of the Supporting Information. 12 compounds with the highest error are provided in Figure S5 of the Supporting Information.

The learning curves of MACE as evaluated on different test sets if given in Figure 3. We see that while the curve seems to be relatively saturated for the ZINC test set, the model’s extrapolation, as measured on the other test sets, is still improving with additional structures. Indeed, the error improves most on the “GDB10–13” test set, which, judging from Figure 4 and discussed in the following section, has a large portion of data points distant from the training set data. Errors on the non-ZINC structures could be improved further by expanding the training set, as a result improving the model’s transferability.

### 3.2 Data Diversity

The performance of MLIPs is closely linked to the diversity of structures within the training data set. The challenge of extrapolation to different data sets is linked to how (dis)similar the structures are to the training data set, in addition to the diversity of the test set structures. In the previous section MACE Performance and Transferability, we showed that MACE extrapolates well to a range of diverse data sets. In this section, we aim to assess the diversity of and relationship between these data sets by calculating geometric descriptors and projecting them into 2D space for visualization.

Local models such as MACE are expected to extrapolate to unseen compounds if all or most of the local environments in the new structure are individually present in the training set, most likely among different structures. This seems to be the case, judging by the global and local SOAP UMAP projections in Figure 4. First, in panel (a), it can be seen that the training set covers virtually all of the test sets in terms of local environments, albeit not uniformly. In contrast, panels (b) and (c) reveal that the test set includes a substantial number of structures distant from the training set, on the outer part of the global SOAP projections. PCA projections and projections of MACE descriptors from Figure S2 of the Supporting Information reveal a similar picture. This suggests that these data sets are testing MACE’s capacity to extrapolate to entirely novel global structures.

Interestingly, most of the structures away from the training set correspond to the GDB subsets of COMP6. This is unsurprising because the GDB database was constructed by enumerating all chemically feasible and synthetically accessible compounds, whereas compounds in the other sets are specifically drug-like. Another curiosity is revealed by coloring the global structure projection according to the magnitude of MACE error, shown in panel (d) of Figure 4 and, for clarity, in Figure S4 of the Supporting Information, where points were plotted according to error in ascending and descending order. Naively, structures further away from the training set might be expected to have high errors. However, some of the structures away from the training set have low errors, and conversely, some of the high-error structures have training set points nearby. 12 of the highest error configurations are depicted in Figure S5 of the Supporting Information. The majority of the structures are from the ”GDB10–13” subset of the COMP6 database; a significant portion of the compounds contain fused rings and/or significantly elongated bonds, particularly the O–H bond. Structures with such significantly elongated O–H bonds are expected to fall outside of the domain of applicability of the MACE model, which was fitted to structures sampled from relatively low-temperature MD simulations. In contrast, the high occurrence of structures with joint rings may be a sign of them being underrepresented in the training set. However, this observation is empirical, based on only 12 structures, some of which have both joined rings and elongated bonds.

### 3.3 Bond Dissociation Energy Prediction

A transferable MLIP trained on neutral molecules and sp^3^ carbon radicals, as explained above, allows us to calculate the homolytic BDEs and radical reaction energies. Accurate BDE estimation is of interest in the context of CYP metabolism prediction, where the relative reactivity of different sites is crucial in determining the site(s) at which a molecule is modified by the enzyme. In this work, we focus on BDEs of aliphatic C–H bonds, which correspond to the hydrogen atom abstraction step of the aliphatic hydroxylation reaction mediated by CYPs.

To calculate a BDE, we first perform geometry optimization of the molecule and the radical obtained by removing the selected hydrogen atom. The BDE is then calculated according to eq 1. The geometry optimization step together with the total energy estimation presents a question of how MACE and DFT BDEs should be compared, which method(s) should be used to optimize the structures on which the BDEs are computed and compared.
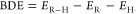
1

**Figure 5:**
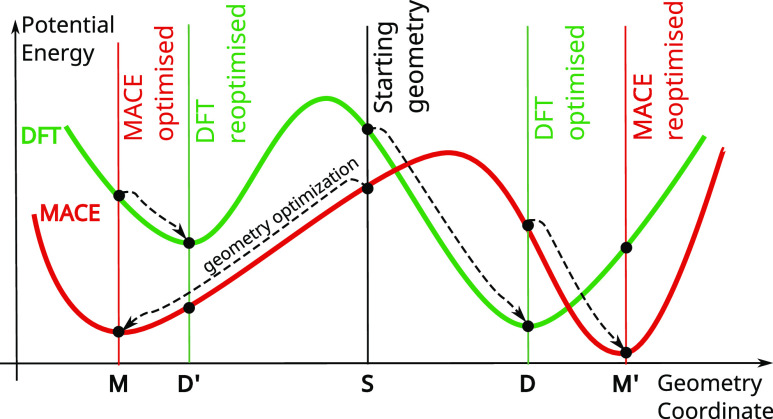
Comparison of the two potential energy surfaces (PESs). **S**: starting geometry, generated by RDKit from SMILES strings, as described in section Data Sets. **M** and **D**: respectively MACE- and DFT-relaxed starting geometry **S**. **D′**: structure obtained by relaxing the MACE-relaxed geometry (**M**) with DFT. **M′**: structure obtained by relaxing the DFT-relaxed geometry (**D**) with MACE.

This problem is equivalent to assessing the wellness of fit between two potential energy surfaces—DFT (reference) and MACE (fitted) in our case. In the first instance, two structures relaxed by the two methods can be compared by computing the root-mean-squared displacement (RMSD) between the atoms’ positions. However, there are several options from which to start geometry optimization with each method, as depicted in Figure 5. Figure 5 illustrates MACE and DFT potential energy surfaces (PESs), both with two local minima that can be roughly matched up. These two minima with a transition state between them may correspond to different conformers that can relatively easily interchange at room temperature, for example. The most obvious way to compare DFT- and MACE-optimized geometries is to relax the same starting geometry with both methods (**S** → **D** and **S** → **M** paths in Figure 5 respectively) and compute RMSD between the resulting structures (**D** and **M**). However, in the case of multiple local minima, as in the engineered example in Figure 5, the minima in which DFT- and MACE-optimized structures end up depends on which minimum’s basin the starting geometry falls under and the chaotic nature of high order optimization methods. Because the choice of the starting geometry is arbitrary, whether the DFT- and MACE-optimized structures fall in the matching local minima is correspondingly starting geometry-dependent. Since geometry optimization is chaotic when using efficient high-order optimizers, even for two closely matched PESs little inconsistencies along the optimization trajectories may lead to nonequivalent local minima. While this is not obvious from the one-dimensional example of Figure 5, this problem is more pertinent to the case of multidimensional optimization, such as 3N-dimensional geometry optimization of an N atom structure. The most efficient path to a local minimum of a many-dimensional surface is not straightforward to determine and different optimization algorithms employ different strategies. As a result, even though the potential energy surfaces of two methods may be practically equivalent, the indeterminate nature of geometry optimization may lead to optimized structures in nonequivalent local minima. Therefore, comparing two methods via a single geometry optimization from the same starting geometry is not the appropriate way to assess the equivalence of two methods for geometry optimization tasks. In the case where MACE and DFT geometry optimization leads to nonequivalent local minima (e.g., **M** and **D** as opposed to the equivalent **M** and **D′** or **M′** and **D**) the large RMSD reflects the separation between these nonequivalent minima instead of the errors in MACE’s approximation to the closest equivalent DFT local minimum. Therefore, care should be taken that only the structures in equivalent DFT and MACE local minima are compared. One approach may be to collect all of the closely related alternative minima for each method by performing multiple geometry optimizations with each method, starting from multiple perturbations of the starting structure **S**. For an error metric that reflects inaccuracies in MACE without the differences in the optimization outcome, BDEs should only be compared by first matching the equivalent minima between the sets of minima obtained by DFT and MACE. However, such an exhaustive DFT-based exploration is significantly computationally expensive.

One straightforward way to ensure that only structures from closest-matched DFT and MACE local minima are compared is to restart the geometry optimization with the second method from a structure already optimized with the first. For example, an arbitrarily chosen starting geometry is optimized with MACE and subsequently, this MACE-relaxed structure is reoptimized with DFT (the **S** → **M** → **D′** path). This procedure ensures that only the closest local minima of the MACE and DFT are compared. Comparing the closest (equivalent) MACE and DFT minima guarantees that only the error of MACE in representing the DFT potential energy surface is reflected in the RMSDs and not the indeterminacy of a starting geometry or the optimization process.

Table 3 summarizes mean RMSDs over the “CYP 3A4” data set (all of the 1177 structures), as computed over different pairs of geometry-optimized structures. First, if strictly equivalent local minima are compared, by means of optimization and reoptimization as discussed above, the mean RMSDs correspond to 0.07 (**D′** vs **M**) and 0.04 (**D** vs **M′**) Å—such a misalignment would be barely noticeable by eye, if at all. The structure mismatch is much larger when both methods’ geometry optimization is started from the same arbitrary structure (0.17 Å for **D** vs **M** geometries), indicating that in at least some of the instances, the structure relaxation with MACE and DFT lead to nonequivalent local minima. This is also supported by similarly large RMSD (0.15 Å) obtained by comparing two sets of DFT-optimized structures: starting geometry directly optimized via DFT (**D**) on one hand and MACE-optimized structure reoptimized with DFT (**D′** local minimum). Finally, we see from the fact that mean RMSD in “**D′** vs **M**” column is larger than that in “**D** vs **M′**” column that optimizing structures with MACE first and then reoptimizing them with DFT leads to local minima somewhat more dissimilar than those obtained by reversing the MACE and DFT optimization order.

**Table 3:** Mean of all Structures’ Positions’ RMSDs (Å) and DFT vs MACE BDE Errors for the “CYP 3A4” Data Set^a^

Geometries	D vs D	D′ vs M	D vs M′	D vs M	D′ vs D
mean RMSD, Å		0.07	0.04	0.17	0.15
BDE RMSE, kcal/mol	1.16	**1.37**	1.24	1.59	1.33
BDE MAE, kcal/mol	0.55	**0.61**	0.59	0.75	0.68
BDE MARE, %	0.64	**0.70**	0.68	0.84	0.76

a RMSD: root-mean-squared displacement, RMSE: root-mean-squared error, MAE: mean absolute error, MARE: mean absolute relative error. Column headers correspond to the two geometries on which DFT and MACE BDEs were correspondingly computed, following notation of Figure 5.

Similar considerations apply when computing MACE’s BDE error with DFT BDEs as the reference because the total energies needed to compute a BDE are evaluated on geometry-optimized structures. A comparison of MACE and DFT BDEs, as computed on geometries relaxed by different methods, is given in Table 3. First of all, RMSE of 1.16 kcal/mol, where both MACE and DFT BDEs are computed on the same (DFT-relaxed, **D**) geometries, reflect the error due to an error in MACE’s single-point evaluation of structures without taking into account the effects of geometry optimization. On the other extreme is the RMSE of 1.59 kcal/mol, where MACE BDEs were calculated on MACE-relaxed starting geometries and DFT BDEs were correspondingly calculated by DFT-relaxing the same starting geometries. As discussed above, this value overestimates the error, because some of the DFT and MACE-relaxed structures correspond to nonequivalent local minima. The most sensibly computed RMSEs correspond to 1.37 and 1.24 kcal/mol, where both DFT and MACE BDEs were computed on DFT and MACE-rerelaxed structures, which ensures that the BDEs are computed and compared on equivalent local minima.

Let us emphasize that in this section we aim to estimate how well MACE approximates the DFT potential energy surface. Therefore, considering strictly equivalent minima allows us to understand how the single-point MACE errors along DFT PES translate to errors in the derived property, the BDE. This comparison also allows us to quantify the size of the effect that the mismatch of the local PES minima has on the overall error. Comparing the strictly matching PES minima is a simplification of comparing the free energies of bond dissociation. Free energy is the most appropriate metric to compare different methods and is preferable to both, matching the local minima (**M** vs **D′** or **M′** vs **D** comparison) and starting the procedure from an arbitrary starting geometry (**D** vs **M**). In computing the free energies, the contributions of different local minima are weighted appropriately, although that significantly increases the computational cost. Ideally, free energies would also be used in applications such as site of metabolism prediction, as illustrated in Figure 1. However, the effect of different conformations on the reaction energies has been found to be small for this application and thus only a single geometry is considered for computational efficiency.^45^

Ultimately, the BDE error of 1.37 kcal/mol is close to the chemical accuracy of 1 kcal/mol that is required for models to be practically useful in realistic applications. As seen from learning curves in Figure 3, MACE accuracy could still be marginally improved by increasing the amount of data it is trained on. However, this result indicates the transferability and usefulness of MACE in predicting BDEs of arbitrary [C,H,O]-containing drug-like compounds and justifies future expansion of the scope of the work to include other chemical elements and reaction mechanisms.

### 3.4 BDE Rank Prediction

While an accurate absolute BDE value prediction is the ultimate goal, an accurate prediction of the order of the BDEs within a given molecule would already be beneficial as an indication of regioselectivity. In the context of the site of metabolism prediction, knowledge of the relative order of metabolism sites could guide further modifications to the proposed structure, for example.

**Figure 6:**
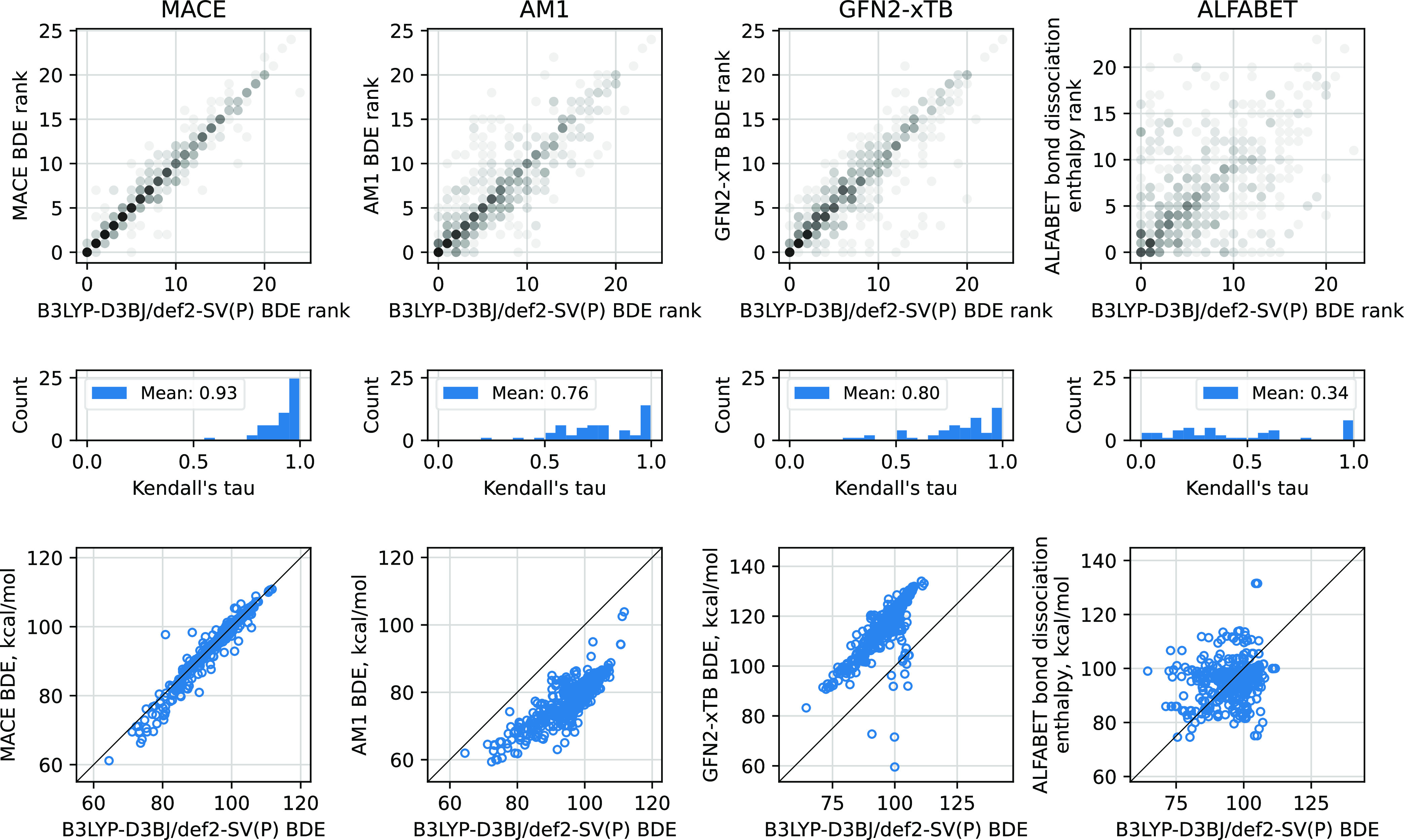
Comparison of MACE, AM1,^47^ GFN2-xTB,^48^ and ALFABET^56^ in their ability to predict BDEs. The top panels show BDE rank correlations for different molecules in the “CYP 3A4” data set. In each plot, multiple BDE rank predictions are overlaid over each other with the resulting gray scale indicating the density of points. The middle graphs show distributions of Kendall’s τ correlation coefficients for all of the molecules in the “CYP 3A4” data set, one τ for one molecule’s BDE rank. In the bottom row are shown parity plots of predicted versus DFT BDE directly. The BDEs are computed on the initial geometries optimized with the respective method (“**D** vs **M**” of Figure 5). The ALFABET model was fitted to reproduce bond dissociation enthalpies computed via M06-2X/def2-TZVP; however, the rank of bond dissociation enthalpies is expected to be comparable to the BDEs used here as the reference.

The top panels of Figure 6 show the predicted BDE rank correlations with respect to the DFT BDE reference. To compare the 60 molecules, we compute BDE ranks with the two methods and plot one rank versus the other for each of the molecules, overlaid over each other in the top row of the plots in Figure 6. The middle row of panels shows the distributions of Kendall’s τ correlation coefficients, each coefficient corresponding to the rank of BDEs of a single molecule. The predicted vs reference BDE parity plots are shown in the bottom row of Figure 6. In addition to MACE, we evaluate two semiempirical methods, AM1^47^ and GFN2-xTB,^48^ and ALFABET,^56^ a graph neural network model created to predict bond dissociation enthalpies of small organic molecules directly based on their 2D SMILES representation.

While different methods (from ab initio to MLIPs to classical force fields) have different shifts for the absolute energy values, BDEs may be directly compared because they rely on energy differences. Similarly, BDE rankings for different molecules are even more directly comparable across different methods, because effects such as nonequivalent local minima discussed in the previous section are less relevant and the ranks are likely to be more consistent among all ab initio methods than the absolute BDE values. Furthermore, the order of C–H bond strengths for a given molecule is expected to be comparable, if not the same, as evaluated in terms of BDEs (property of interest in this work) and bond dissociation enthalpies (property predicted by the ALFABET model). Therefore, to compare different methods in their ability to predict BDEs, we primarily consider the distribution of correlation coefficients (middle panels) and the quality of the BDE rank correlations, including any outliers, in the top panels of Figure 6. The correlation of BDEs is included in the bottom row of panels in order to get a sense of outliers and general trends, rather than expecting a perfect correlation in the cases of AM1, GFN2-xTB, and ALFABET.

First, in Figure 6, we see that the MACE BDE ranks correlate with those of DFT well, with few BDEs predicted significantly out-of-order, as indicated by the few off-diagonal outliers. The mean correlation coefficient is correspondingly high, and the distribution of these correlation coefficients is peaked toward the value of 1.

Both of the semiempirical methods show similar performance, with GFN2-xTB having a slightly better correlation of the two. Both of the methods’ rank correlations show more outliers than that of MACE. The mean correlation coefficients are lower than that of MACE, but similar for both of the semiempirical methods—AM1 (0.76) and GFN2-xTB (0.80). The parity plot of GFN2-xTB shows a similarly offset correlation as that of AM1, where the BDEs are generally overpredicted by GFN2-xTB and under-predicted by AM1. Furthermore, the GFN2-xTB outliers are due to an oxygen-stabilized radical (see Figure S7 of the Supporting Information), however, it is unclear whether the outliers are an artifact of GFN2-xTB or are missed by the reference DFT. Disregarding this offset and outliers, the spread of the bulk of the GFN2-xTB predictions is tighter than the spread of the AM1 predictions, as reflected in the higher mean correlation coefficient for the former.

Finally, ALFABET’s bond dissociation enthalpy predictions appear to exhibit a negligible correlation with the DFT BDEs. The fact that ALFABET’s predictions target a different DFT approach [M06-2X/def2-TZVP, not B3LYP-D3BJ/def2-SV(P)] and compute enthalpies, not energies, render direct comparison inappropriate. The bottom right panel in Figure 6 is included for completeness. In this plot, a correlation might appear if B3LYP enthalpies were plotted instead of energies because the points on the plot coming from different molecules may be shifted by different amounts due to different sizes of the thermal and zero-point energy contributions for molecules of different sizes. However, for a given molecule and derived radicals used to calculate the C–H BDEs these corrections are expected to be similar, and therefore, the BDE ranks are expected to correlate with bond dissociation enthalpy ranks in the top-left panel of Figure 6. We speculate this poor rank correlation is because the compounds in the “CYP 3A4” data set are out of the domain of applicability of the ALFABET model due to their size and/or the chemical motifs present in the compounds. The training set of ALFABET contains bond dissociation enthalpies computed for compounds with up to 9 heavy atoms, whereas the largest compound in the “CYP 3A4” data set has 42 atoms; see Figure S1 of the Supporting Information for full compound size distribution. Therefore, we reproduce the DFT BDE and ALFABET bond dissociation enthalpy comparison of Figure 6 on 55 compounds with up to 9 heavy atoms; see Figure S6 of the Supporting Information. On these data, the BDE/enthalpy rank correlation has few significant outliers and the majority of the sp^3^C–H bonds are assigned the correct position in the rank within one place. Furthermore, the direct BDE versus energy comparison (bottom panel of Figure S6 of the Supporting Information) now reveals an offset correlation, not present in the equivalent plot of larger structures in the bottom right panel of Figure 6.

### 3.5 Extrapolation to Nonequilibrium Structures

While prediction of equilibrium properties, such as reaction or BDEs, is the aim of this work, access to nonequilibrium structures and derived properties, would allow one to study a much wider range of phenomena. In the context of metabolism prediction, reaction activation energies are expected to offer a much better description of the lability toward CYP metabolism than BDEs do.

**Figure 7:**
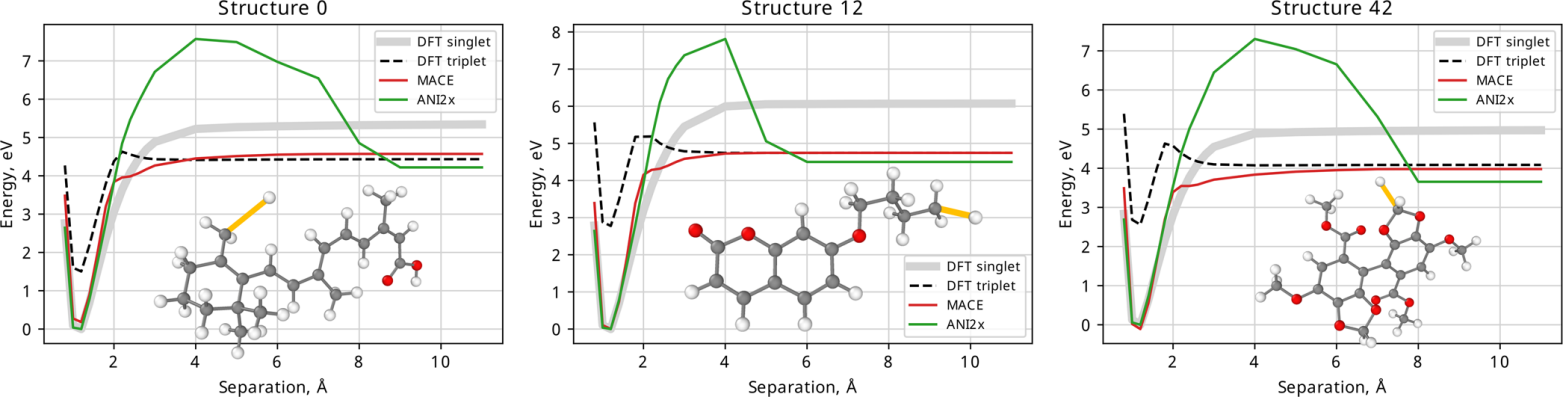
MACE, ANI-2x, and DFT predictions as the highlighted bond of an optimized structure is stretched. The DFT and ANI-2x scales have been shifted for the lowest point to correspond to 0. MACE curves were shifted by the same amount as the DFT curves.

In Section MACE Performance and Transferability, we show that the MACE model extrapolates well to the compounds distinct from those in the training set. Figure 7 shows the results of an extrapolation test to nonequilibrium structures, with more examples found in Figure S8 of the Supporting Information. In this test, we take molecules from the “CYP 3A4” data set, change the distance between selected C–H atoms, and evaluate them with MACE, DFT, and ANI-2x.

Generally, when fitting MLIPs, the aim is to represent the ground state potential energy surface. In the case of DFT, the lowest energy state changes from singlet to triplet as a bond is stretched, at around 2–3 Å in Figure 7. The MACE model has only been fitted to the near equilibrium structures; its training set contains structures corresponding to small separation (below approximately 2 Å, closed-shell molecules) and large separation (above approximately 5 Å, open-shell radicals) in Figure 7 but not in between. While MACE is not expected to reproduce the kink at around 2–3 Å because that region of PES is not present in its training set, MACE interpolates smoothly between the singlet and the triplet states. It is desirable for the MLIP models to behave smoothly and regularly in the regions removed from the structures in its training set. While ANI-2x gives a reasonable prediction for the radical energy, the dissociation path has a large and unreasonable barrier. Although the molecule is kept rigid during this bond scan, this smooth transition in MACE is encouraging for future extensions to predictions of nonequilibrium, reactive structures.

### 3.6 Hydrogen Abstraction Minimum Energy Paths

While the main focus of the article is on modeling near-equilibrium closed- and open-shell structures, extending this work to describe the full MEPs would be the next most impactful follow-up. Specifically in the context of BDE and site of metabolism prediction, reaction activation energies are expected to better correlate with the reaction rates and, consequently, to be a better predictor of the metabolite ratios. To demonstrate the feasibility of such a development, we slightly extend the initial training set and apply it to predict MEPs of methoxy radical-mediated hydrogen abstraction. The methoxy radical is a known simplified system for the direct estimation of the activation energy for the hydrogen abstraction reaction.^79^

**Figure 8:**
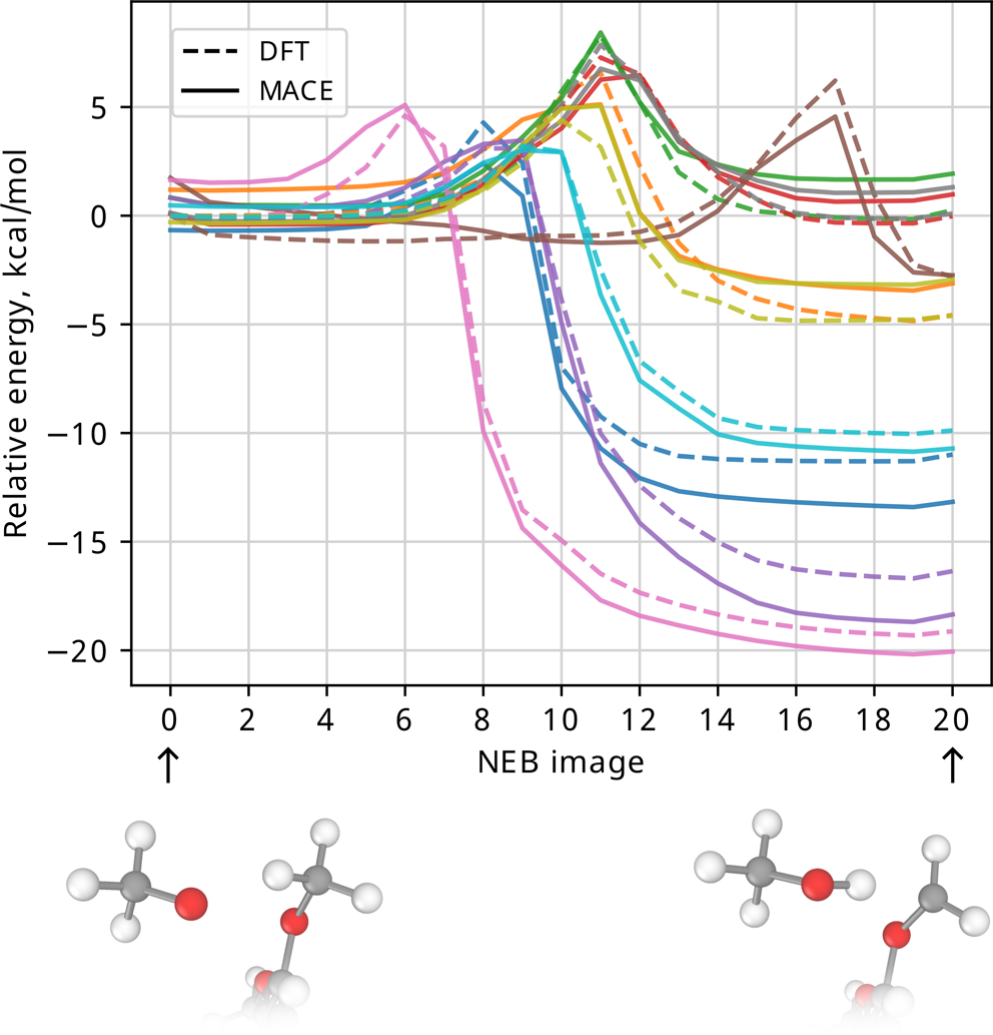
MEPs of hydrogen abstraction by methoxy radical of 10 compounds (different colors), as determined by MACE_NEB_ and re-evaluated with DFT. All paths have been plotted with respect to the DFT energy of the first structure in the MEP. The insets below the figure illustrate the end points of the MEPs. Note that none of the 10 compounds and no structures along the MEP have been included in the MACE_NEB_ training set.

To create the MACE_NEB_ model, we took a subset of the full MACE training set, described in section Train and Test Sets, and extended it with some structures necessary for a nudged elastic band (NEB) calculation.^80^ The two principal components of this extension were isolated methanol molecule and methoxy radical configurations and molecule-methoxy radical and radical-methanol molecule pairs, with the methanol molecule or methoxy radical positioned appropriately for removal of the hydrogen atom from a sp^3^C–H bond. This constituted the MACE_NEB_ training set of 4089 structures, out of which roughly a third were new NEB-related additions—see Section II of the Supporting Information for a full description. The MACE_NEB_ interatomic potential was applied to find MEPs of hydrogen removal of 50 new molecules. Out of the 50 simulations, 33 NEB calculations have converged with the maximum force below 0.1 eV/Å (approximately 2.3 kcal/mol). The 10 lowest error paths are plotted in Figure 8 with the RMSE of 35.1 kcal/mol/at, as evaluated over all the structures in these paths.

This is another extrapolation test because no structures in the middle region of the hydrogen abstraction path have been included in the training set of MACE_NEB_, only the reagent and product geometries. However, with only isolated molecules, radicals, and reagent and product complexes in its training set, the MACE_NEB_ potential is able to yield reasonable converged MEPs, even if to a relatively high maximum force threshold of 0.1 eV/Å and high error. Both the NEB convergence and accuracy along the MEPs may be improved by expanding the MACE_NEB_ training set with novel compounds (c.f. learning curves of Figure 3) and nonequilibrium reactive structures from along the full hydrogen abstraction path. Active learning approaches, where new structures are added based on some model uncertainty or predicted error criterion, would be helpful for faster model error convergence.^41,81−83^ Active learning has already been applied to iteratively improve MACE models which were used to calculate NEB-based reaction barriers, however, in the context of heterogeneous catalysis (e.g., for hydrogenation of carbon dioxide to methanol over indium oxide^35^) and not gas-phase small molecule reactions.

## 4 Conclusions

In this paper, we have demonstrated that the MACE framework may be used to construct a transferable MLIP for closed-shell drug-like molecules and selected derived radical species. We have shown that for the closed-shell structures, the MACE model reaches accuracy on par with a general-purpose transferable ANI-2x MLIP but can, in addition, describe selected open-shell structures to similar accuracy. One explanation for the success in transferability is that the training set constructed in this work covers a wide range of local chemical environments. This suggests that the ZINC database is a good source of structures on which to base MLIP fitting frameworks and MD-based geometry sampling was sufficient to achieve high transferability. Moreover, the MACE framework seems to be well suited for reaching high accuracy at a relatively low training data-regime limit of fewer than 20,000 structures. Furthermore, the MACE model shows promise in an extrapolation test to nonequilibrium structures.

The MACE model’s applicability to open-shell structures allowed us to predict BDEs of interest because of their relevance in cytochrome P450 metabolism prediction. We have shown that MACE matches the reference DFT values within RMSE of 1.16–1.59 kcal/mol, with the most appropriate comparison leading to a RMSE of 1.37 kcal/mol. In addition, the order of BDEs within a molecule is predicted better with MACE than it is with a semiempirical AM1 method, commonly used for high-throughput BDE and site of metabolism predictions,^84,85^ a more recent GFN2-xTB semiempirical method^48^ or an alternative ALFABET model for predicting the bond dissociation enthalpies.^56^

Overall, this study showcases the potential of MLIPs in modeling structures beyond near-equilibrium or closed-shell compounds. Going toward a method to replace semiempirical methods, such as commonly used AM1, in metabolism modeling, the current work would need to be extended to include more chemical elements than carbon, hydrogen, and oxygen. Furthermore, the model would need to be extended to cover more mechanisms of hydroxylation reaction, such as the addition of the oxidizing radical species to the substrate. Finally, reaction activation energies are expected to be better indicators of lability toward CYP metabolism, and for this MACE would need to be extended to cover the full reaction path. The feasibility of such an approach has been demonstrated by extending the MACE model with reactants and products of hydrogen abstraction by methoxy radical reactions, with the resulting model capable of finding reasonable full MEPs.

## Supporting Information Available

Structures in the “ZINC-train” and “ZINC-test” data sets; compounds in the “CYP 3A4” data set; and the MACE model (ZIP)
Further details on the generation of MACE and MACE_NEB_ training sets, key MACE model parameters, distributions of compound sizes in different data sets, details on the 2D projections, additional 2D projections (including MACE descriptors and PCA), high error structures, ALFABET BDE predictions of smaller compounds, GFN2-xTB BDE outliers, and additional C–H bond dissociation curves (PDF)
