# Supplementary material for: Transferable Machine Learning Interatomic Potential for Bond Dissociation Energy Prediction of Drug-like Molecules
Source: J Chem Theory Comput. 2023 Dec 18;20(1):164–77. doi: 10.1021/acs.jctc.3c00710 (PMC10782450; doi:10.1021/acs.jctc.3c00710)
Supplement: Supplementary file 2 — ct3c00710_si_002.pdf [file ct3c00710_si_002.pdf]

Transferable machine learning interatomic potential for bond dissociation energy prediction of drug-like molecules - supporting information

Elena Gelžinytė and Gábor Csányi
Engineering Laboratory, University of Cambridge,
*Trumpington Street, Cambridge CB2 1PZ, United Kingdom**

Mario Öeren and Matthew D. Segall
Optibrium Limited, Cambridge Innovation Park, Denny End Road, Cambridge CB25 9GL, United Kingdom

I. TRAIN AND TEST SET GENERATION PROCEDURE

As described in the main text, to construct the training set, "drug-like" SMILES strings were downloaded from the ZINC database[5], from which 3D geometries of the closed-shell molecule structures were generated with RDKit[3]. These served to additionally create multiple open-shell structures by in turn removing a hydrogen atom from the sp^3C-H bonds of the parent closed-shell molecule. From this pool of closed- and open-shell structures, 16824 were selected as the starting configurations for the molecular dynamics (MD) simulations at 500 K temperature. From each of the MD trajectories, a single structure was selected and re-evaluated with the reference DFT method to constitute the "final" training set.

The "final" training set was constructed in three stages. First, the MD simulations were performed with the semi-empirical GFN2-xTB method[1] on 2798 closed- and open-shell structures (a subset of the above 16824). One structure was selected from each of the MD trajectories and re-evaluated with the reference DFT method to yield an "initial" training set. The "initial" MACE model was fitted to these initial data. Next, the MD simulations were performed on an additional 7010 closed- and open-shell structures (a different subset of the above 16824). This time, however, the "initial" MACE model was used to drive these simulations, instead of the semi-empirical GFN2-xTB. A single structure from each of the 7010 MD trajectories was selected, evaluated with the reference DFT and added to the structures of the "initial" training set to yield the "second" training set of 9808 closed- and open-shell structures. The "second" MACE model was fitted to these data and used as above to drive the MD simulations, from which an additional 7016 structures were selected, re-evaluated with the reference DFT method and added to the "second" training set. Altogether, this has yielded the "final" training set with the 16824 structures described above and in Table 1.

The "initial" and "second" MACE models were only used to drive the MD simulations for expanding the "initial" training set and are not discussed in the main text. Similarly, the "initial" and "second" training sets appear only in Figure 3, where the "final" MACE is re-evaluated on the corresponding sub-sets of the "final" training set in order to produce a learning curve.

A justification for using the "initial" and "second" MACE models to drive the MD simulation, is to gather structure samples from a slightly-incorrect MACE Boltzmann distribution. The inclusion of these structures in the training set of the subsequent MACE model, now with the correct reference DFT energies and forces, should bring the MACE-MD sampled distribution closer to that of the reference DFT. In such active learning schemes, the initial ML models may yield unstable MD simulations, especially if applied to structures outside of their training set. To catch such failures, the MD trajectories were monitored by tracking the number of neighbours of all atoms in the structure and ensuring the correct valency: hydrogen atoms were allowed strictly one neighbour, oxygen – one or two, carbon – two, three or four. Only structures from intact MD trajectories and with all force components below 15 eV/Å were included among the final 16824, with the "failed" trajectories corresponding to only a few percent of the total number of the performed MD simulations. We did not explicitly monitor for fragmentation or rearrangement reactions, however, only transformations that did not violate the allowed neighbour count and had reasonable DFT force components were included in the "final" training set, e.g. see compound No. 10 in Figure S5.

An equivalent procedure was used to generate the ZINC test set, started from entirely new [C,H,O]-containing SMILES strings. The only difference from the procedure used to generate the training set, is that for the test set the MD simulations were run at 300 K temperature, with the GFN2-xTB method only. For evaluation of MACE error, energies and forces of structures in this test set were re-evaluated with the B3LYP-D3BJ/def2-SV(P) reference method

described in Section Reference method. The MD simulations for the training set were run at a higher temperature than the equivalent MD simulations of the test set structures (500 K instead of 300 K) in order to sample more diverse training set structures and extend the range of MLIP applicability slightly beyond the intended test MD temperature.

II. MACE$_{\text{NEB}}$ TRAINING SET

The training set of MACE$_{\text{NEB}}$ described in Section Bond dissociation energy prediction has been built on the "initial" training set (2798 closed- and open-shell structures) described in the above Section I, but by extending it with structures needed to initialise a nudged elastic band (NEB) minimum energy path calculation. The additional structures included 122 methanol and methoxy radical configurations (sampled from 500 K GFN2-xTB MD) and 986 molecule + methoxy radical and radical + methanol molecule pairs. These pairs were obtained by first optimising the molecule or radical in isolation and then placing a methoxy radical or methanol molecule at an appropriate distance and orientation with respect to the hydrogen atom of an sp^{3}C-H bond. (In the context of NEBs, these structures would be further geometry-optimised to obtain reactant and product geometries to serve for the initial NEB path generation.)

III. MACE PARAMETERS

Key parameters used to fit the MACE models described in the main test are reported in Table S1. The exact version of the MACE fitting code used in this work may be found at `https://github.com/gelzinyte/mace/tree/mace100323`.

Parameter	Value	Explanation
r_{cut}	5 Å	Cutoff
T	2	Number of interactions
ν	3	Correlation order
L	3	Max L
k	128	Number of channels
`start_swa`	500	Loss scheduler at epoch

TABLE S1: Key MACE parameters. Notation in the first column follows Reference[2] or MACE documentation at `https://github.com/ACEsuit/mace`. Note that r_{cut} of 5 Å with two interactions ($T = 2$) yields an effective cutoff of 10 Å.

IV. DISTRIBUTIONS OF NUMBER OF ATOMS IN DIFFERENT DATASETS

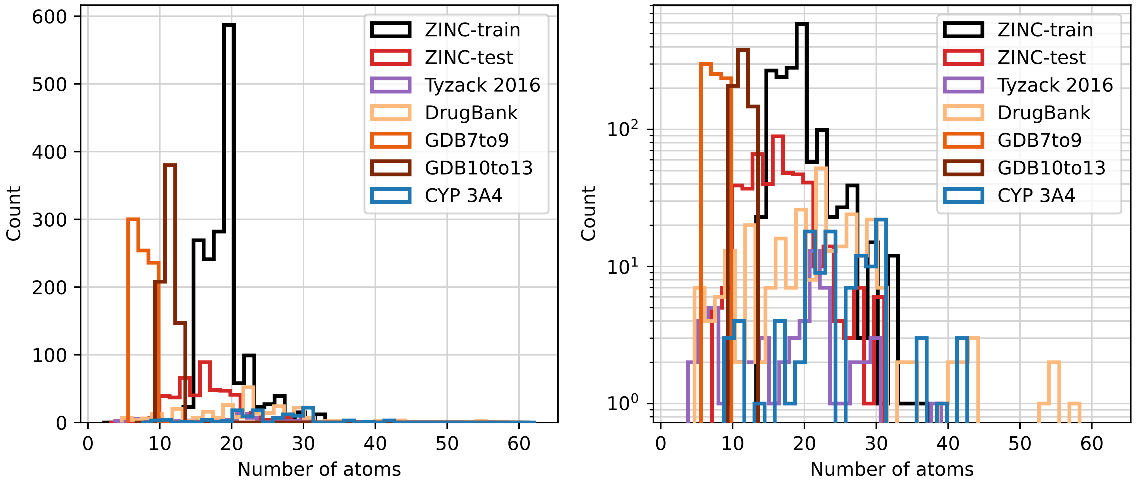

FIG. S1: Distribution of the number of heavy atoms in compounds from various datasets described in Table 1. The y-axis is plotted in linear scale on the left and logarithmic scale on the right.

V. 2D PROJECTION DETAILS

In addition to UMAP projections of local and global SOAP descriptors, here we report PCA projections and MACE descriptors.

As well as the SOAP descriptor, we evaluate the structures with the MACE descriptors. SOAP descriptor is independent of data, while the MACE descriptor is part of the MACE model and is optimised to the training data, so the descriptors complement each other. The local MACE descriptor is formed from the learnt node features concatenated across the layers. All descriptor instances were normalized to have unit norm.

To project these descriptors we used Principal Component Analysis (PCA) and Uniform Manifold Approximation and Projection (UMAP)[4]. PCA projects data to a 2D space defined by orthogonal directions that capture most of the variance in the data. In contrast, UMAP is a non-linear projection method that aims to preserve the global structure of the data. More details and UMAP parameters are given in Section V B

A. SOAP parameters

Parameter	Value	Explanation
r_{cut}	6.0 Å	Local environment cutoff
σ_{a}	0.3 Å	Atom Gaussian width
$n_{\max}$	12	Number of radial channels
$l_{\max}$	6	Number of angular channels

TABLE S2: Parameters used to generate SOAP descriptors.

B. UMAP parameters

For the UMAP projection, we set `n_neighbors=25` (neither local nor global structure is strongly preferred) and `min_dist=0.001` (points in 2D representation are allowed to be packed closely). The following metric was used to define distance (D) between two descriptors ($\mathbf{d}_1$ and $\mathbf{d}_2$):

$$D = \sqrt{2 - 2 \times \mathbf{d}_1 \cdot \mathbf{d}_2} \quad (1)$$

VI. 2D PROJECTIONS

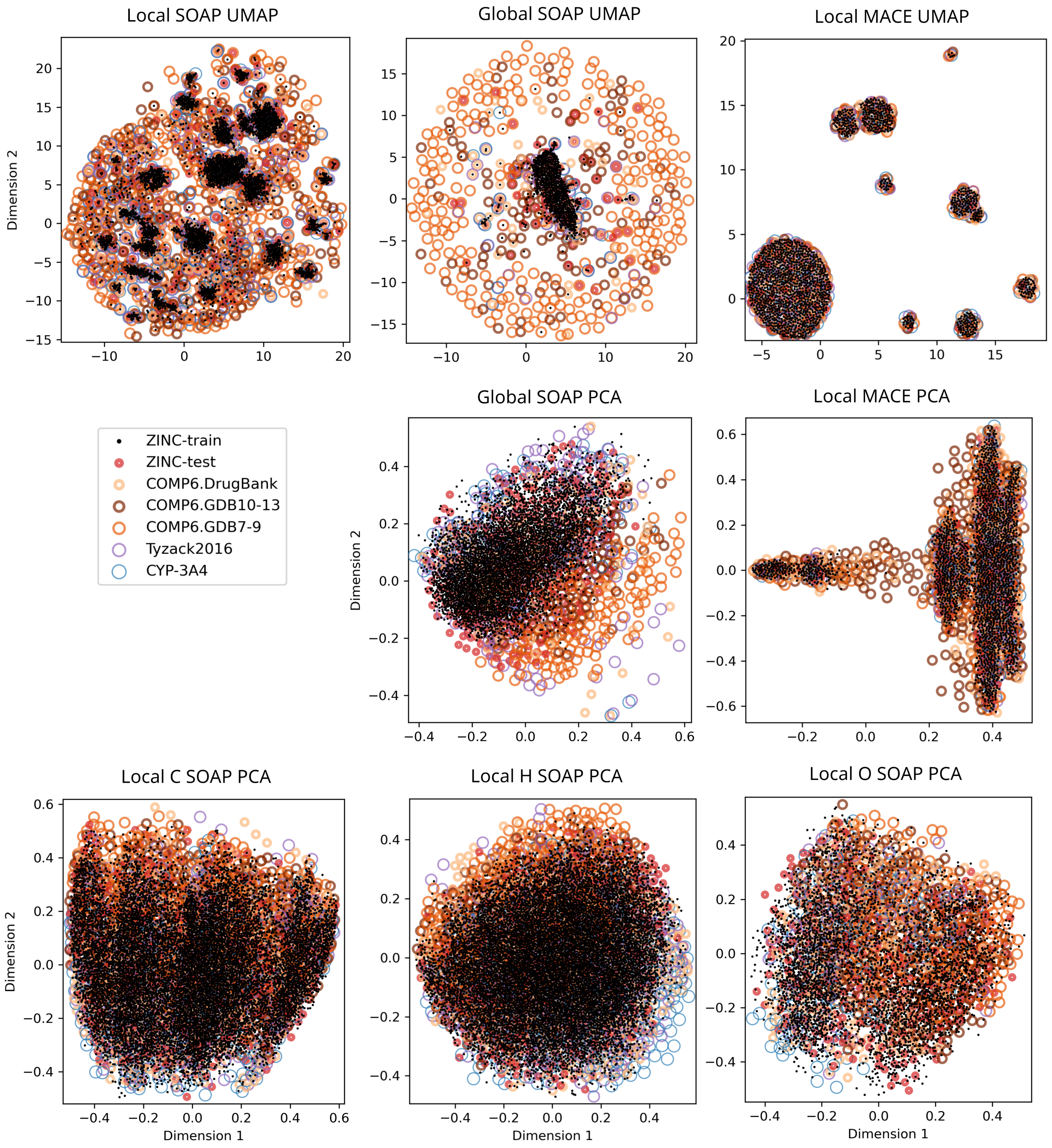

FIG. S2: (local SOAP, global SOAP, local MACE) x (UMAP, PCA) projections. For clarity, the overlapping data points were sparcified within each of the datasets. For computational tractability, only a tenth of the local environments were projected for MACE UMAP and PCA and local SOAP PCA.

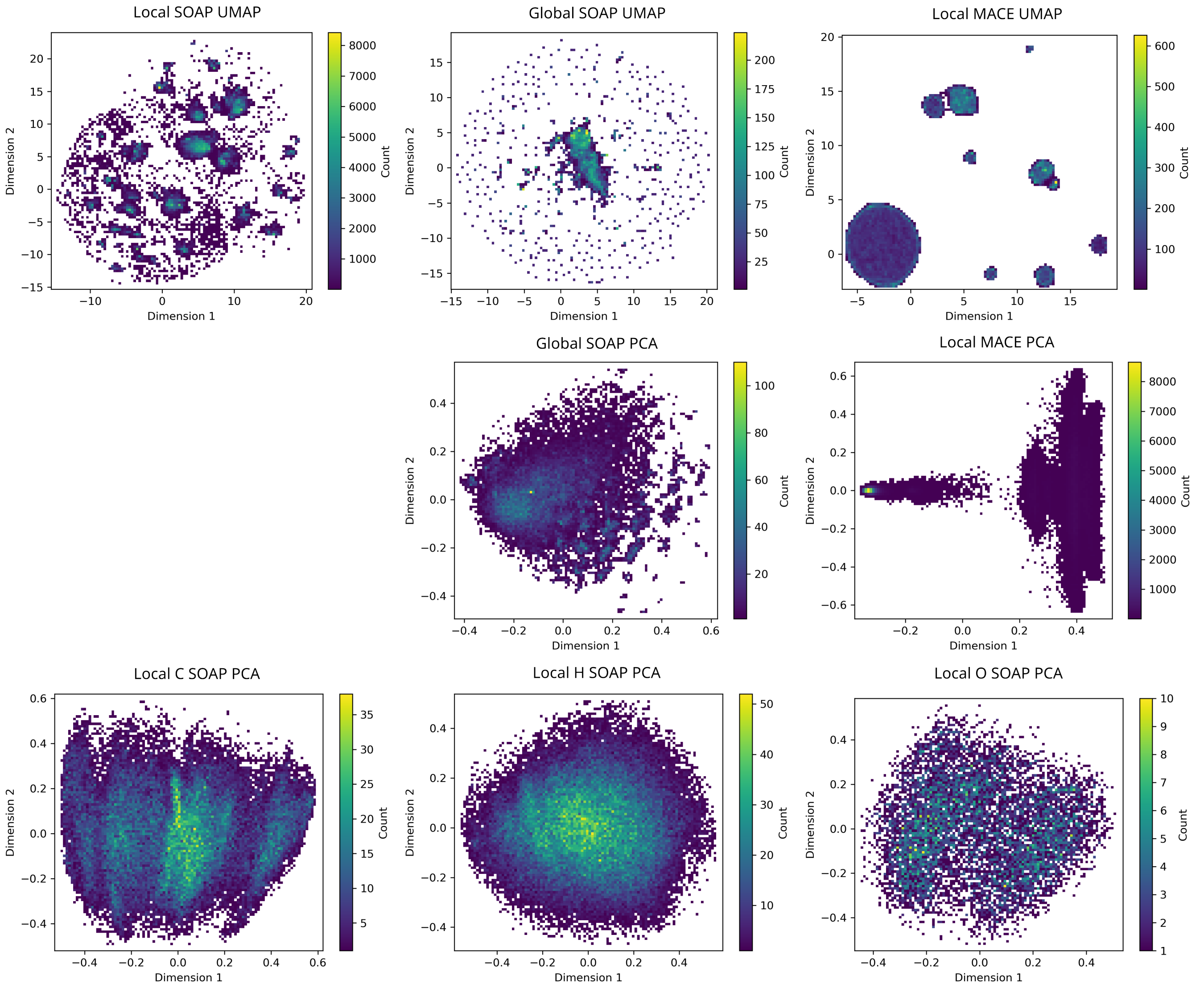

FIG. S3: Density of points in (local SOAP, global SOAP, local MACE) x (UMAP, PCA) projections. For computational tractability, only a tenth of the local environments were projected for MACE UMAP and PCA and local SOAP PCA.

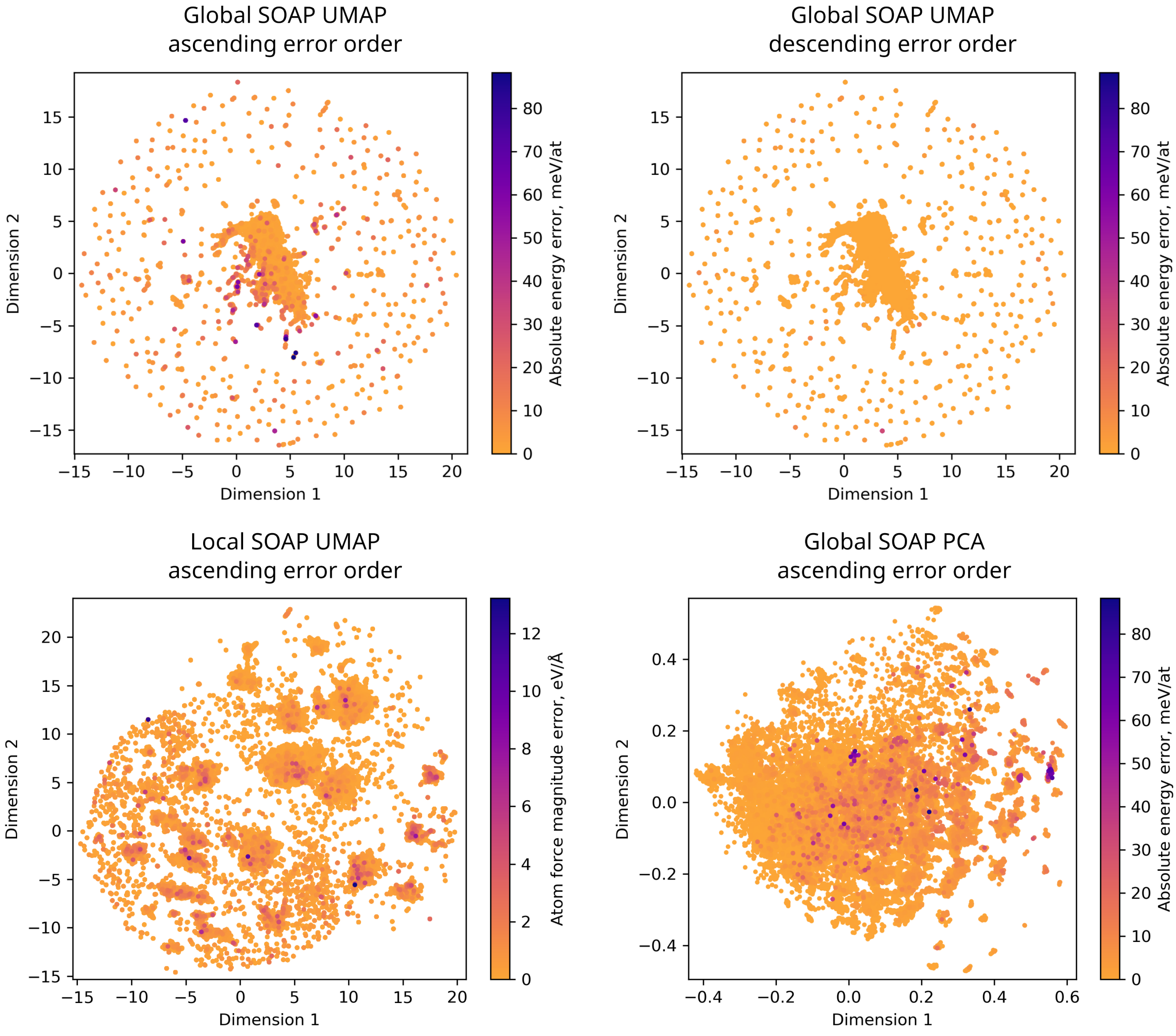

FIG. S4: local SOAP UMAP, global SOAP UMAP and global SOAP PCA projections coloured by error. The points from the bottom layer to the top layer are ordered either in ascending or descending order of error. Only the points from test sets, not the "ZINC-train" dataset are shown.

VII. HIGH ERROR OUTLIERS

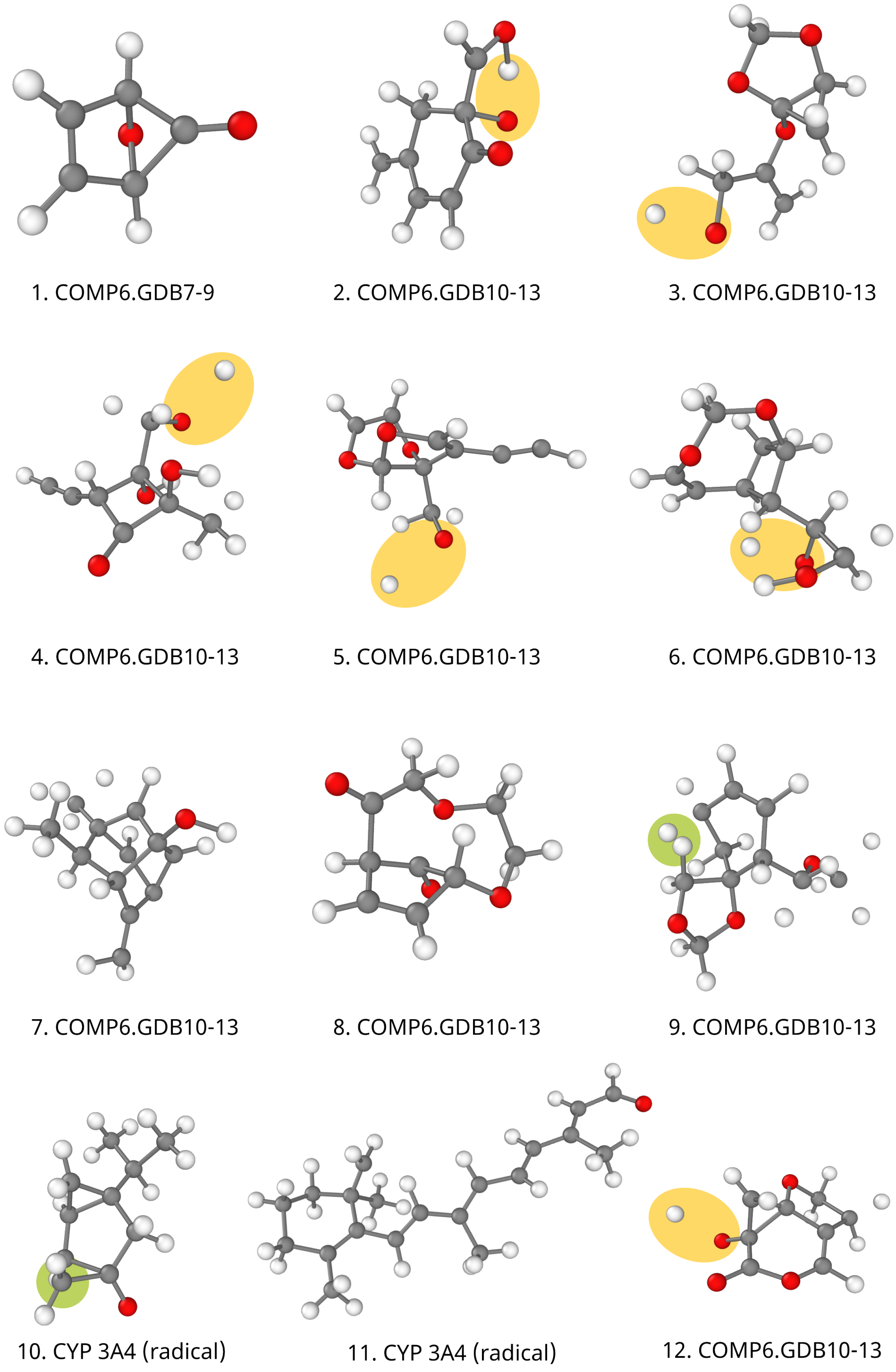

FIG. S5: 12 structures with the highest error in Figure 4. Most of them have multiple rings, significantly elongated C-H (not rendered) and O-H (highlighted in yellow) bonds, and a close approach (Structure 9, highlighted). Structure 10 (sampled from GFN2-xTB MD trajectory) is an open-shell structure that had a hydrogen atom originally removed from the highlighted carbon atom which subsequently was stabilised by creating the lower 3-member ring.

VIII. ALFABET BDES OF SMALLER COMPOUNDS

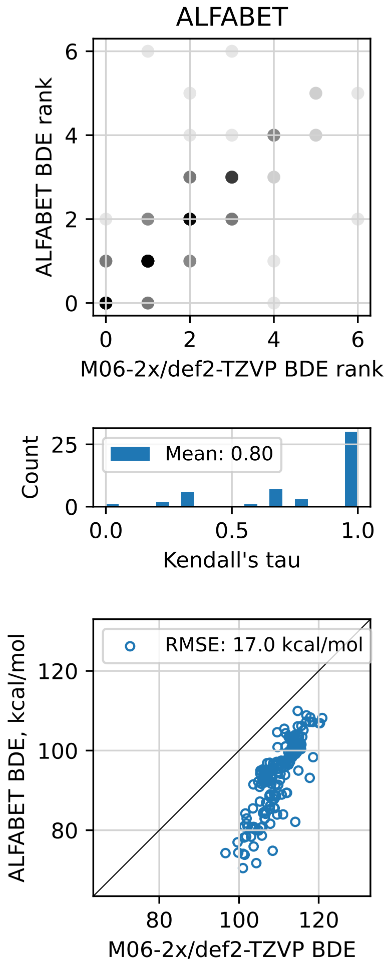

FIG. S6: Top: ALFABET sp^3C-H bond dissociation enthalpy ranks vs DFT bond dissociation energy ranks for 55 compounds from the "COMP6.GDB7-9" dataset. Middle: distribution of Kendall's τ coefficients. Bottom: ALFABET bond dissociation enthalpy vs DFT bond dissociation energy parity plot.

IX. GFN2-XTB BDE OUTLIERS

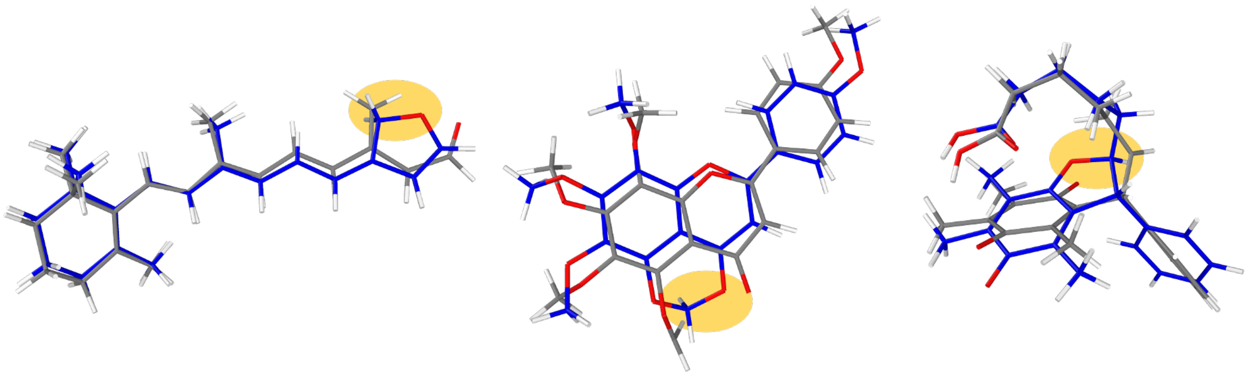

FIG. S7: Three open-shell structures corresponding to the compounds with the lowest GFN2-xTB BDEs in Figure 6. DFT-optimised (B3LYP-D3BJ/def2-SV(P)) geometries are in grey and GFN2-xTB-optimised in blue. All three of the structures have the radical carbon (from which the hydrogen atom was removed) stabilised by a nearby oxygen atom. This additional bond is highlighted in yellow.

X. BOND DISSOCIATION CURVES

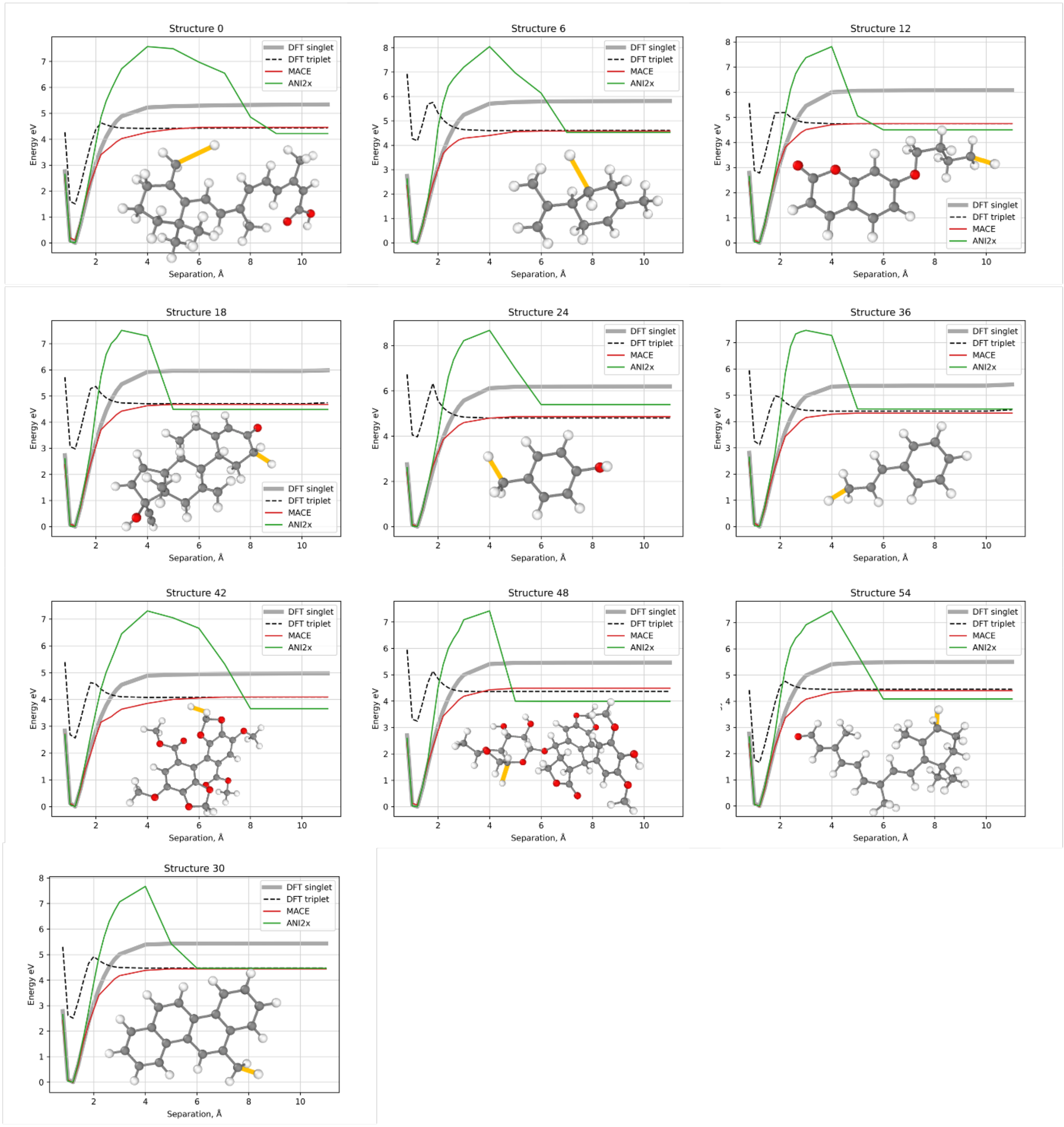

FIG. S8: Additional structures with C-H bond dissociation curves.

* Electronic address: eg475@cam.ac.uk

[1] Christoph Bannwarth, Sebastian Ehlert, and Stefan Grimme. Gfn2-xtb—an accurate and broadly parametrized self-consistent tight-binding quantum chemical method with multipole electrostatics and density-dependent dispersion contributions. *Journal*

of chemical theory and computation, 15(3):1652–1671, 2019.

[2] Ilyes Batatia, David P Kovacs, Gregor Simm, Christoph Ortner, and Gábor Csányi. Mace: Higher order equivariant message passing neural networks for fast and accurate force fields. *Advances in Neural Information Processing Systems*, 35:11423–11436, 2022.

[3] Greg Landrum. Rdkit: Open-source cheminformatics. https://www.rdkit.org.

[4] Leland McInnes, John Healy, and James Melville. Umap: Uniform manifold approximation and projection for dimension reduction. *arXiv preprint arXiv:1802.03426*, 2018.

[5] Teague Sterling and John J Irwin. Zinc 15–ligand discovery for everyone. *Journal of chemical information and modeling*, 55(11):2324–2337, 2015.
